# Validation of Vetscan Imagyst^®^, a diagnostic test utilizing an artificial intelligence deep learning algorithm, for detecting strongyles and *Parascaris* spp. in equine fecal samples

**DOI:** 10.1186/s13071-024-06525-w

**Published:** 2024-11-12

**Authors:** Ashley Steuer, Jason Fritzler, SaraBeth Boggan, Ian Daniel, Bobby Cowles, Cory Penn, Richard Goldstein, Dan Lin

**Affiliations:** 1 School of Veterinary Medicine, Texas Tech University, 7671 Evans Drive, Amarillo, TX 79106 USA; 2 Zoetis Inc, 10 Sylvan Way, Parsippany, NJ 07054 USA; 3 Zoetis Inc, Veterinary Medicine Research and Development, 333 Portage St, Kalamazoo, MI 49007 USA; 4 Analitix Giant Clinical Research Co., LTD, Commercial Center Bldg 1, 258 Lvdi Avenue, Huaqiao, Kunshan, Suzhou, 21532 China

**Keywords:** Artificial intelligence, Deep learning, Diagnostic, Equine, Fecal egg, *Parascaris*, Sheather’s sugar solution, Strongyles

## Abstract

**Background:**

Current methods for obtaining fecal egg counts in horses are often inaccurate and variable depending on the analyst’s skill and experience. Automated digital scanning of fecal sample slides integrated with analysis by an artificial intelligence (AI) algorithm is a viable, emerging alternative that can mitigate operator variation compared to conventional methods in companion animal fecal parasite diagnostics. Vetscan Imagyst is a novel fecal parasite detection system that uploads the scanned image to the cloud where proprietary software analyzes captured images for diagnostic recognition by a deep learning, object detection AI algorithm. The study describes the use and validation of Vetscan Imagyst in equine parasitology.

**Methods:**

The primary objective of the study was to evaluate the performance of the Vetscan Imagyst system in terms of diagnostic sensitivity and specificity in testing equine fecal samples (*n* = 108) for ova from two parasites that commonly infect horses, strongyles and *Parascaris* spp., compared to reference assays performed by expert parasitologists using a Mini-FLOTAC technique. Two different fecal flotation solutions were used to prepare the sample slides, NaNO_3_ and Sheather’s sugar solution.

**Results:**

Diagnostic sensitivity of the Vetscan Imagyst algorithm for strongyles versus the manual reference test was 99.2% for samples prepared with NaNO_3_ solution and 100.0% for samples prepared with Sheather’s sugar solution. Sensitivity for *Parascaris* spp. was 88.9% and 99.9%, respectively, for samples prepared with NaNO_3_ and Sheather’s sugar solutions. Diagnostic specificity for strongyles was 91.4% and 99.9%, respectively, for samples prepared with NaNO_3_ and Sheather’s sugar solutions. Specificity for *Parascaris* spp. was 93.6% and 99.9%, respectively, for samples prepared with NaNO_3_ and Sheather’s sugar solutions. Lin’s concordance correlation coefficients for VETSCAN IMAGYST eggs per gram counts versus those determined by the expert parasitologist were 0.924–0.978 for strongyles and 0.944–0.955 for *Parascaris* spp., depending on the flotation solution.

**Conclusions:**

Sensitivity and specificity results for detecting strongyles and *Parascaris* spp. in equine fecal samples showed that Vetscan Imagyst can consistently provide diagnostic accuracy equivalent to manual evaluations by skilled parasitologists. As an automated method driven by a deep learning AI algorithm, VETSCAN IMAGYST has the potential to avoid variations in analyst characteristics, thus providing more consistent results in a timely manner, in either clinical or laboratory settings.

**Graphical Abstract:**

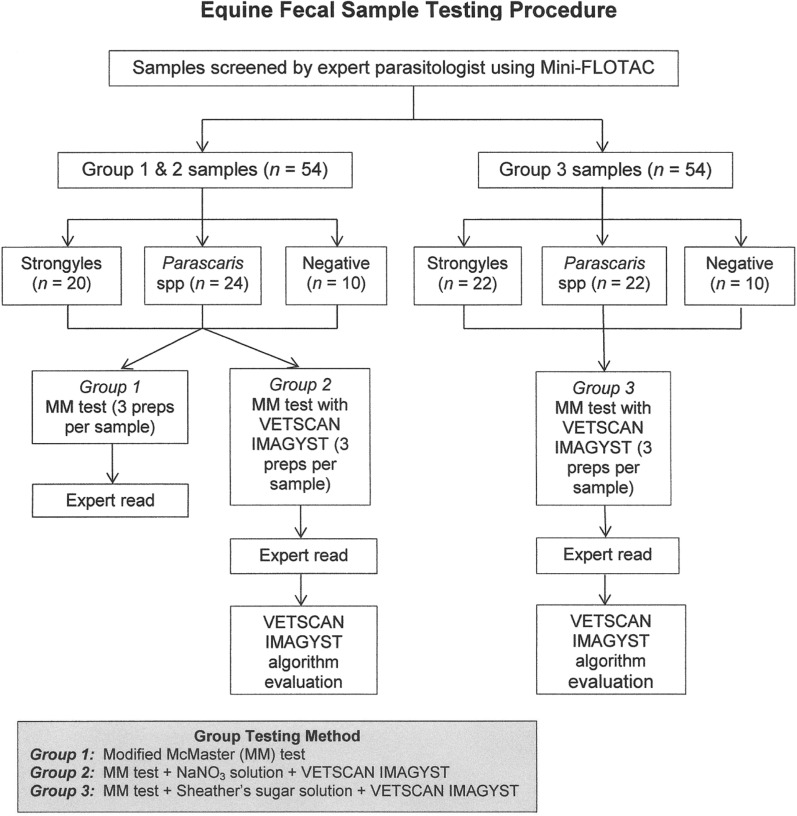

## Background

Fecal egg count (FEC) is the principal diagnostic tool for equine parasite management. A FEC helps determine a deworming regimen suitable for the herd or individual horses, including the efficacy of antiparasitic treatment. The latter application is particularly relevant considering that resistance to various classes of equine anthelmintics is a persistent problem in clinical practice [1–3]. Fecal egg count is also useful for identifying horses that are inherent high-shedders and warrant individual management [3].

Despite its status as the mainstay for nematode diagnosis in horses, the accuracy of the FEC test as traditionally performed can be quite variable depending on the skill and experience of the person reading the cytology slides [2, 4–6]. Variations in sample preparation methods can also affect FEC diagnostic sensitivity [1, 2, 7]. The impact of these limitations was illustrated in a FEC diagnostic study (*n* = 335 FEC tests) at the University of Pennsylvania, where veterinary parasitologists concluded that fecal flotation examinations in companion animal practice could be reasonably expected to miss up to half of infected patients because of test variability or technician error [8]. Other investigators found that variations in FEC technical proficiency occur even when personnel follow identical methods and sets of directions [4].

Automated digital scanning of fecal sample slides integrated with analysis by an artificial intelligence (AI) algorithm is emerging as a viable alternative to conventional microscopic evaluation methods [2, 5, 9–15]. An automated AI approach has the potential to improve diagnostic accuracy by minimizing variations in individual skills and experience of personnel performing the FEC test and subjective microscopic interpretation of fecal samples. In other words, diagnostic interpretation shifts from an individual technician to an AI algorithm. Digital data from individual cases can be transmitted to an internet cloud (i.e., servers dedicated to the diagnostic application) where it is used to continuously refine the morphologic profile of the target parasite. Several reviews on the potential contributions of AI to laboratory diagnostic accuracy and efficiency have been recently published [12, 16, 17].

Vetscan Imagyst (Zoetis Services LLC, Parsippany, NJ, USA) is a novel fecal parasite detection system in horses consisting of a modified McMaster (MM) sample preparation technique and an automated digital scanner that uploads the scanned image to the cloud where proprietary software analyzes captured images for recognition by a deep learning, object detection algorithm. Once analysis of the scanned image is completed, the algorithm then assigns the scanned image to a specific parasite genus that the software has been trained to recognize. Analysis of the scanned slide can generally be completed within 10 to < 15 min, with minimal need for trained expertise [6, 18]. As the Vetscan Imagyst algorithm evaluates additional visual data, it can distinguish the morphology of specific parasite ova on fecal flotation slides with increasing accuracy [6, 18]. The system also has the quantitative ability to perform a FEC for the target parasite. Developed expressly for use in veterinary practice, the Vetscan Imagyst system is an all-in-one, multi-use platform that can be used in clinical, point-of-care, or laboratory settings.

Studies of Vetscan Imagyst performance in diagnosing fecal parasite ova in dogs and cats have been previously reported [6, 18]. This is the first published report describing its use in horses. The primary objective of the study described here was to validate the performance of the Vetscan Imagyst system in terms of diagnostic sensitivity (correctly determining the percentage of samples that were true positives) and specificity (correctly determining the percentage samples that were true negatives) in testing equine fecal samples for ova from two target parasites.

## Methods

### Study site and personnel

Fecal sample preparation and analyses were performed at the Parasitology Laboratory at the Texas Tech University School of Veterinary Medicine in Amarillo, Texas, USA. Parasitologists who performed fecal sample preparation and evaluation were blinded to the individual identity of fecal samples evaluated either by manual prescreening or the Vetscan Imagyst algorithm. Components of the system were supplied by the manufacturer to laboratory personnel, who were trained in using the platform specifically for testing and evaluating equine fecal samples.

### Vetscan Imagyst testing system

The components of the Vetscan Imagyst system have been previously described in some detail [6, 18]. Laboratory equipment included an Ocus 40 digital slide scanner (Grundium Ltd, Tampere, Finland) and Apacor coverslips and sample transfer loops (Apacor Ltd, Wokingham, UK). Briefly, the algorithm identifies the most discriminating features of target parasites in a fecal sample preparation, beginning when the entire prepared slide is scanned and progressing to pixel-level recognition. The scanned image is then broken down into smaller images, called scenes. The scenes are further evaluated and broken down into convolutional blocks. At this level the pixels of the images are converted to differentiating features, such as shape, edge, color gradient, or certain configuration edges in a deeper layer of the network. The process is continuously repeated to create simpler, more abstract features. The features from the last convolutional block are suitable for classification and object detection. After all the features have been extracted for the diagnostic sample, they are utilized to calculate a probability score for target parasite ova. Only images above a specified threshold are reported. The identity and image of the target parasite are posted to an online platform that can be accessed with a web browser.

### Sample preparation and evaluation

The study evaluated qualitative and quantitative coprodiagnostic outcomes for two of the most pervasive and clinically relevant equine intestinal parasites, strongyles (cyathostomins) and *Parascaris* spp. [19–22]. A total of 108 freshly collected fecal samples were obtained from naturally infected foals and adult horses at equine farms in North Texas, Oklahoma, and Kentucky in the USA. Refrigerated samples were stored for no more than 2 weeks. Each sample was submitted with individual animal clinical data utilizing a standardized form supplied with the study protocol. At least 50 g of equine feces per sample was required for inclusion in the study. All fecal samples used in the study were initially prescreened by an expert parasitologist using the Mini-FLOTAC test with a salt or sugar flotation solution (SG 1.26) to confirm the presence of strongyle or *Parascaris* spp. ova or negative parasite status. Copromicroscopy methods used in the Mini-FLOTAC assays have been previously described [23]. Features used to identify the target parasite eggs are illustrated in a standard reference [24]. Two flotation solutions were evaluated with the systems algorithm, sodium nitrate NaNO_3_ (SG 1.22) and Sheather’s sugar solution (SG 1.26).

Results of the Mini-FLOTAC prescreening testing were the gold standard for the parasite count for each sample. The eggs per gram (EPG) count was determined by counting the eggs in the grid of each slide chamber and multiplying the total by 5.

#### Fecal sample preparation

**Fig. 1:**
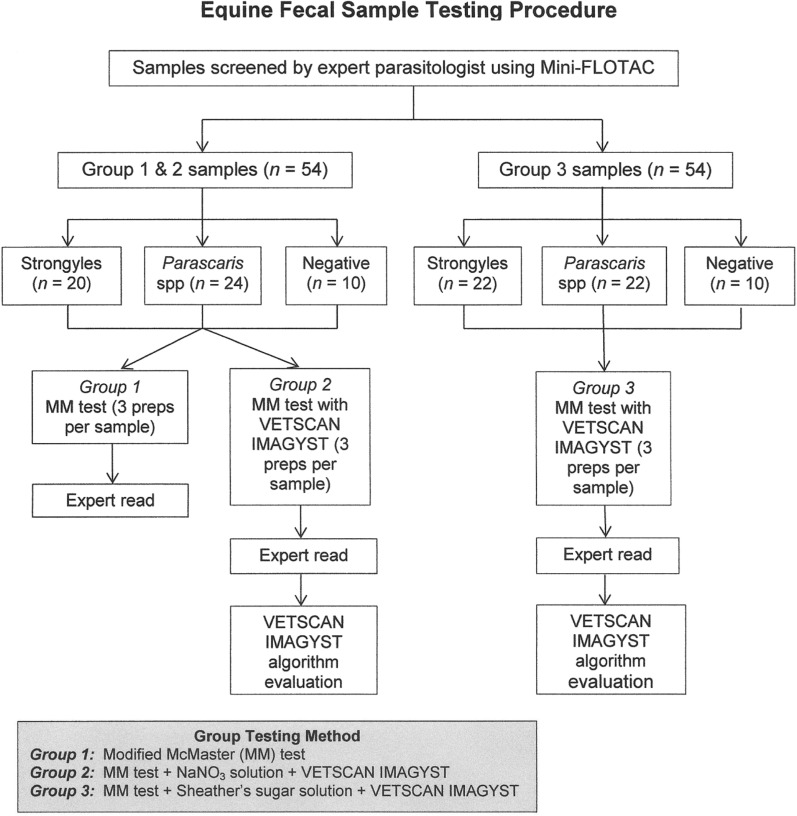
Equine fecal sample testing procedure. Sample collection and analysis procedures for evaluating equine fecal samples using the Vetscan Imagyst testing system. All samples were from naturally infected horses. Three different testing methods were used to evaluate samples for target parasites: Group 1, modified McMaster (MM) test; Group 2, MM test + NaNO_3_ flotation solution evaluated with Vetscan Imagyst algorithm; Group 3, MM test + Shearer’s sugar flotation solution with Vetscan Imagyst algorithm.

Preparation for Group 1 was done with a MM prep method. For this process, 4 g of feces was measured into a clean labeled disposable cup and mixed with 26 ml flotation solution (NaNO_3_). The feces was then mixed into the solution well for 30–60 s and filtered through two-ply cheesecloth into a clean labeled disposable cup. After mixing, the solution was immediately loaded into a McMaster chamber slide for evaluation by the parasitology expert and then prepared for Vetscan Imagyst analysis (see Fig. 1).

### Modified McMaster slide preparation and evaluation

Once the two-chamber McMaster slide was loaded, it was allowed to set for 30 s before evaluation. Using 100 × magnification, both grids of the chambers were counted, added together, and then multiplied by an factor of 25 to result in an EPG per sample.

#### Vetscan Imagyst slide preparation and evaluation

The fecal solution filtrate described above was used for Vetscan Imagyst slide preparation after the McMaster Sample had been removed and utilized. After the concentration step, an Apacor transfer loop was used to obtain a sample of the flotation solution, which was placed onto a glass slide. A specialized coverslip with fiducials was then placed over the flotation sample on the slide for purposes of scanning (see Fig. 1). First, the slide was analyzed by a parasitologist, and all the parasite ova under the coverslip were counted before being analyzed by the Vetscan Imagyst.

#### Samples processed

Positive samples for either target parasite were included in the study until 42 strongyle-positive, 46 *Parascaris* spp.-positive, and 20 negative samples had been processed. Samples testing as positive could include either or both strongyles and *Parascaris* spp. Of the 108 samples, 49 (45.4%) were positive for both strongyles and *Parascaris* spp., including 29 coinfections in samples processed with NaNO_3_ solution and 20 coinfections processed with Sheather’s sugar solution. Each sample was uniquely numbered and classified by FEC density (low, medium, high) for strongyle and *Parascaris* spp. ova, respectively, as shown in Table 1.

**Table 1:** Eggs per gram (EPG) ranges for target parasites in equine fecal samples

Parasite and EPG range	EPG	No. samples
Strongyles		
Negative	0	10
Low	5–199	15
Medium	200–500	14
High	> 500	13
*Parascaris* spp.		
Negative	0	10
Low	1–99	13
Medium	100–300	19
High	> 300	14

### Study design

The primary objective of the study was to assess the performance in terms of sensitivity and specificity of the Vetscan Imagyst equine algorithm for the target parasite ova compared to reference assays performed by expert parasitologists using MM copromicroscopy methods under laboratory conditions. Test results were compared for fecal samples prepared with either of two flotation solutions, NaNO_3_ or Sheather’s sugar solution. Precision of the algorithm was determined by comparing results of triplicate assays for each fecal sample. Performance was also assessed in comparison to results obtained from Mini-FLOTAC prescreening of fecal samples. A secondary objective was to qualitatively and quantitatively assess how the algorithm identifies and classifies ova of the two target parasites compared to test results obtained by expert parasitologist under laboratory conditions.

The prescreened equine fecal samples (with mini-FLOTAC) were randomly assigned by a consulting biometrician to one of three study groups (Fig. 1). Group 1 samples were manually evaluated by an expert parasitologist using a passive-flotation modified McMaster (MM) test using a sodium nitrate (NaNO_3_) flotation solution. Group 2 and Group 3 samples were prepared using the methods above and evaluated manually by an expert parasitologist using one of two different fecal flotation solutions, followed by evaluation with Vetscan Imagyst. Group 2 samples were prepared as described above using NaNO_3_ solution and then evaluated with the system. Group 3 samples were prepared as described above using Sheather’s sugar solution and then evaluated. To determine test precision, slides were prepared and evaluated in triplicate for each sample within each group. The expert parasitologist evaluated the sample slides qualitatively and quantitatively for the target parasite ova in a randomized order for each sample preparation. A sample was considered positive if any eggs were observed.

All three study groups included the same number of total fecal samples (54). The same set of 54 fecal samples was used for Groups 1 and 2, while a separate set of 54 fecal samples was used for Group 3 (Fig. 1). Group 1 and 2 samples were positive for strongyles (*n* = 20 samples) or *Parascaris* spp. (*n* = 24) or had negative parasite status (*n* = 10). Group 3 samples were positive for strongyles (*n* = 22) or *Parascaris* spp. (*n* = 22) or had negative status (*n* = 10).

Parasite recovery performance of the Group 1 MM test and Groups 2 and 3 Vetscan Imagyst tests for the target parasites was compared to corresponding results of the Mini-FLOTAC prescreening test. Sensitivity and specificity of the systems algorithm for the target parasites in relation to the MM reference test performed manually by an expert parasitologist were calculated for the Group 2 and 3 samples.

Agreement of manual EPG counts by the expert parasitologist for the triplicate slide preparations per sample within each study group was calculated using logarithmic transformation values for the EPG counts and expressed as a coefficient of variation (CV). Agreement of the EPG counts for Group 2 and 3 samples as determined by the automated algorithm was calculated using logarithmic transformation values and expressed as a CV. A correlation scatterplot was constructed to graphically illustrate the performance of the quantitative EPG count for Group 2 and 3 samples versus the expert parasitologist’s EPG count.

Agreement between manual EPG count and automated Vetscan Imagyst EPG counts was expressed as Lin’s concordance correlation coefficient (CCC). Taken together, the various components of the study determined the accuracy of the expert parasitologist’s qualitative and quantitative evaluations of EPG for the target parasites in equine fecal samples and determined the sensitivity and specificity of the systems qualitative or quantitative analysis to two reference methods, the Mini-FLOTAC prescreening assays and the MM test, both administered by expert parasitologists.

### Data analysis

Positive agreement between the AI algorithm result and the expert parasitologist’s assessment was defined as ≥ 95% agreement. The Mini-FLOTAC reading was the gold standard for positive diagnosis and FEC and was the value against which each MM test result was compared. Sensitivity and specificity with a 95% confidence interval considering the three replicates for each sample were calculated using the bivariate generalized linear model for binomial proportions. Accuracy of the MM test by expert analysis and analysis by Vetscan Imagyst were evaluated using a bivariate regression model for the repeated measure positive counts with a logarithmic transformation by using MM test results by expert counts as the reference.

## Results

### Diagnostic sensitivity vs. pre-screening reference test

Diagnostic sensitivity of the three test methods compared to results of the pre-screening Mini-FLOTAC reference test is shown in Table 2. All three sample preparation methods correctly identified positive strongyle samples in ≥ 99.9% of the cases. *Parascaris* spp. sensitivity ranged from 67.3% for the MM test + NaO_3_ solution evaluated by VETSCAN IMAGYST (Group 2) to 99.9% for the MM test + Sheather’s sugar solution evaluated by VETSCAN IMAGYST (Group 3).

**Table 2:** Sensitivity of testing methods in detecting target parasite eggs in equine fecal samples vs. prescreening with Mini-FLOTAC reference test

Sample population and target parasite	Test method and % sensitivity (95% CI)		
	Group 1 (modified McMaster test)	Group 2 (modified McMaster test + NaO_3_ solution + Vetscan Imagyst)	Group 3 (modified McMaster test + Sheather’s sugar solution + Vetscan Imagyst)
All samples			
Strongyles	99.9% (99.9–100%)	99.9% (99.9–100%)	99.9% (99.9–100%)
*Parascaris* spp.	95.3% (85.7–100%)	67.3% (47.4–90.1%)	99.9% (99.9–100%)
Samples excluding those with low EPG counts^a^			
Strongyles	99.9% (99.9–100%)	99.9% (99.9–100%)	100% (100%)
*Parascaris* spp.	99.9% (93.8–100%)	77.5% (55.8–99.9%)	98.3% (95.0–100%)

^a^Strongyle samples with < 25 EPG, *Parascaris* spp. samples with < 15 EPG

There was a notable difference in sensitivity of Group 2 and Group 3 assays in relation to the Mini-FLOTAC prescreening test. While the sensitivity of Group 2 and 3 tests for strongyles was equivalent, the sensitivity percentage of the Group 2 test for *Parascaris* spp. was nearly a third less than the percentage for the Group 3 test (Table 2). When sensitivity of the systems algorithm was compared with tests performed by expert parasitologists, the Group 2 tests had lower specificity for strongyles (91.4% vs. 99.9%), lower sensitivity (88.9% vs. 99.9%) for *Parascaris* spp., and lower specificity (93.6 vs. 99.9%) for *Parascaris* spp. compared to Group 3 tests (Table 4). Precision of testing methods for both target parasites generally favored Group 3 methods (Tables 3 and 5).

When positive samples with low parasite EPG counts (strongyle EPG < 25, *Parascaris* spp. EPG < 15) were excluded, diagnostic sensitivity was unchanged for strongyles and marginally improved for *Parascaris* spp. (Table 2). For example, *Parascaris* spp.-positive results for Group 2 increased from 67.3% for all samples to 77.5% positive results when samples with EPG counts < 15 were excluded.

In summary, compared to the Mini-FLOTAC reference method, all three sample preparation methods had comparable sensitivity performance for strongyles, and exclusion of low-EPG samples did not affect results. Sensitivity for detection of *Parascaris* spp. was > 95% for Group 1 and 3 samples and least favorable for Group 2 samples (67.3%). *Parascaris* spp. sensitivity for Group 2 samples improved somewhat when low-EPG samples were excluded, but *Parascaris* spp. sensitivity was still lower than that for Group 3 samples.

### Precision of human expert diagnosis of target parasites

The CV was lower for all test groups as EPG density increased (Table 3). The Group 3 samples had a lower CV for strongyles versus the other two groups at all EPG levels, and a lower CV for *Parascaris* spp. for high-density samples (EPG > 300). For samples with low EPG counts (5–200 EPG), Group 3 samples had a much lower CV for strongyles than the other two groups, 13.1% vs. 26.7% (Group 1) and 32.8% (Group 2).

**Table 3:** Precision of equine fecal egg counts determined by expert parasitologist expressed as a coefficient of variation (CV)

Target parasite and EPG count range	Intra-group CV for each sample preparation method^a^		
	Group 1 (modified McMaster test) (%)	Group 2 (modified McMaster test + NaNO_3_ solution + Vetscan Imagyst) (%)	Group 3 (modified McMaster test + Sheather’s sugar solution + (Vetscan Imagyst) (%)
Strongyles (5–200 EPG)	26.7	32.8	13.1
Strongyles (201–500 EPG)	5.6	5.4	3.7
Strongyles (> 500 EPG)	2.6	3.7	0.4
*Parascaris* spp. (5–100 EPG)	26.5	17.7	26.4
*Parascaris* spp. (101–300 EPG)	20.5	20.7	11.4
*Parascaris* spp. (> 300 EPG)	5.0	9.6	0.7

^a^EPG counts calculated as logarithmic transformation values

### Algorithm sensitivity and specificity vs. expert testing

The Vetscan Imagyst algorithm’s performance was assessed by comparing the sensitivity and specificity of the expert parasitologist’s analysis of the sample slide versus the systems algorithm’s analysis of the same slide (Table 4). The algorithm’s diagnostic sensitivity versus manual testing for strongyles was 99.2% (95% CI 97.7–100%) and 100% (95% CI 100–100%) for Groups 2 and 3, respectively. Diagnostic sensitivity for *Parascaris* spp. was 88.9% (95% CI 81.4–99.3%) and 99.9% (95% CI 99.9–100%) for Groups 2 and 3, respectively. Diagnostic specificity for strongyles ranged from 91.4% (95% CI 82.9–100%) for Group 2 to 99.9% (95% CI 99.9–100%) for Group 3. Diagnostic specificity for *Parascaris* spp. was 93.6% (95% CI 89.6–99.5%) and 99.9% (95% CI 99.9–100%) for Groups 2 and 3, respectively. For both target parasites, the systems diagnostic results closely matched those of the expert parasitologist.

**Table 4:** Sensitivity and specificity of Vetscan Imagyst algorithm vs. expert parasitologist in identifying target parasite eggs in equine fecal samples

Target parasite and diagnostic category	Sample preparation method and mean diagnostic accuracy % (95% CI)	
	Group 2 (modified McMaster test + NaNO_3_ solution + Vetscan Imagyst)	Group 3 (modified McMaster test + Sheather’s sugar solution + Vetscan Imagyst)
Strongyles		
Sensitivity	99.2% (97.7–100%)	100.0% (100–100%)
Specificity	91.4% (82.9–100%)	99.9% (99.9–100%)
*Parascaris* spp.		
Sensitivity	88.9% (81.4–99.3%)	99.9% (99.9–100%)
Specificity	93.6% (89.6–99.5%)	99.9% (99.9–100%)

### Algorithm precision in quantifying target parasites

The intra-group precision of the Vetscan Imagyst algorithm in quantifying parasite ova in Groups 2 and 3 is shown in Table 5. The CV for strongyle EPG counts, expressed as logarithmic transformation values, was appreciably lower for the Group 3 triplicate samples versus the Group 2 samples, indicating relatively less dispersion in EPG counts. The CV for *Parascaris* spp. was comparable between Groups 2 and 3 for the low-EPG EPG samples but was appreciably lower for the Group 3 medium- and high-EPG samples, indicating that the Group 3 sample preparation method provides less variable EPG counts across samples, particularly as EPG density increases.

**Table 5:** Precision of Vetscan Imagyst algorithm in identifying target parasite eggs in equine fecal samples expressed as a coefficient of variation (CV)

Target parasite and EPG count range	Intra-group CV for each sample preparation method^a^	
	Group 2 (modified McMaster test + NaNO_3_ solution + Vetscan Imagyst) (%)	Group 3 (modified McMaster test + Sheather’s sugar solution + Vetscan Imagyst) (%)
Strongyles (5–200 EPG)	13.6	9.3
Strongyles (201–500 EPG)	4.3	2.7
Strongyles (> 500 EPG)	2.2	0.3
*Parascaris* spp. (5–100 EPG)	28.5	32.1
*Parascaris* spp. (101–300 EPG)	30.2	19.8
*Parascaris* spp. (> 300 EPG)	17.1	0.8

^a^EPG counts calculated as logarithmic transformation values

### Correlation of algorithm quantitative evaluation with expert analysis

**Fig. 2:**
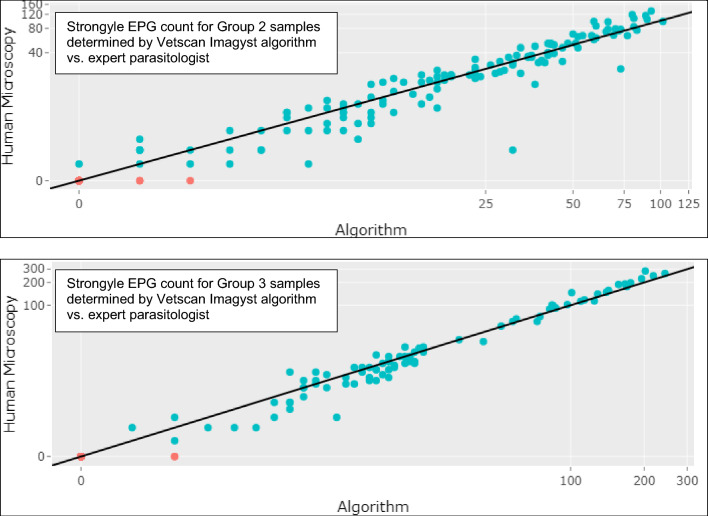
Comparison of strongyle EPG count determined by Vetscan Imagyst and Expert Parasitologist. Algorithm performance versus an expert parasitologist’s strongyle EPG count is shown as a correlation scatterplot where EPG from the Vetscan Imagyst assay was plotted on the x-axis and EPG from the expert parasitologist on the y-axis. The closer the data points come to the straight diagonal line, the greater the correlation between the two testing methods. The algorithm’s performance improved for both test methods as EPG counts increased. Top: Lin’s concordance correlation coefficient (CCC) for Group 2 samples was calculated as 0.924, indicating moderate agreement. Bottom: Lin’s CCC for Group 3 samples was calculated as 0.978, indicating substantial agreement

**Fig. 3:**
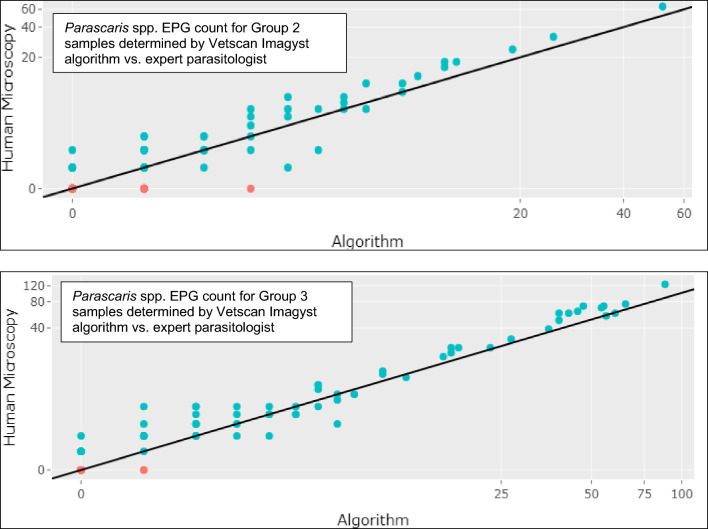
Comparison of *Parascaris* spp. EPG count determined by Vetscan Imagyst and Expert Parasitologist. Algorithm performance versus an expert parasitologist’s *Parascaris* spp. EPG count is shown as a correlation scatterplot where EPG from the Vetscan Imagyst assay was plotted on the x-axis and EPG from the expert parasitologist on the y-axis. The closer the data points come to the straight diagonal line, the greater the correlation between the two testing methods. The algorithm’s performance improved for both test methods as EPG counts increased. Top: Lin’s concordance correlation coefficient (CCC) for Group 2 samples was calculated as 0.955, indicating substantial agreement. Bottom: Lin’s CCC for Group 3 samples was calculated as 0.944, indicating moderate agreement

Correlation scatterplots were created (Figs. 2 and 3) where the EPG data for the algorithm were plotted on the x-axis and the EPG data from the expert parasitologist were plotted on the y-axis. For strongyle EPG counts, Lin’s CCC for Group 2 samples was 0.924, indicating moderate agreement between the Vetscan Imagyst results and the expert analysis. Lin’s CCC for Group 3 strongyle samples was 0.978, indicating substantial agreement. For *Parascaris* spp. EPG counts, Lin’s CCC for Group 2 samples was 0.955, indicating moderate agreement. Lin’s CCC for Group 3 *Parascaris* spp. samples was 0.944, indicating moderate agreement.

### EPG multiplier factor

The MM test multiplier for strongyles was 34.1 and 25.0 for samples from Groups 2 and 3, respectively. The multiplier for *Parascaris* spp. was 66.6 and 36.2 for samples from Groups 2 and 3, respectively.

## Discussion

The various components of the study offer compelling evidence that a Vetscan Imagyst system can consistently provide diagnostic accuracy equivalent to manual evaluations by skilled parasitologists. Sensitivity of the systems algorithm was the key performance indicator for its ability to accurately detect presence of strongyles and *Parascaris* spp. in naturally infected fecal samples, including those with low EPG counts. The sensitivity of for strongyles was comparable to the widely used Mini-FLOTAC and MM reference tests performed manually by expert parasitologists. As an automated method driven by a deep learning AI algorithm, Vetscan Imagyst has the potential to avoid variations in analyst characteristics, thus rendering more consistent results in a timely manner, either at the point of service or in clinical or laboratory settings. The systems testing procedure is efficient, with the time required to complete all steps of an on-site evaluation of a fecal sample typically requiring < 10 min [18].

Detection of parasite ova is generally less assured when the fecal sample contains a low number of eggs [4, 5, 18]. In such cases, marginally infected horses may escape detection if sample collection fails to recover eggs or if copromicroscopy fails to detect widely or unevenly distributed eggs that are actually present in the sample. When Vetscan Imagyst results were compared to the Mini-FLOTAC prescreening test, sensitivity for strongyles was 99.9% for both Groups 2 and 3 whether or not samples with low EPG counts (< 25 EPG) were included. Similarly, for Group 3 samples, sensitivity of the Vetscan Imagyst test for *Parascaris* spp. was little affected when EPG counts < 15 EPG were excluded from the analysis (98.3% vs. 99.9% for all samples). These results indicate that the VETSCAN IMAGYST algorithm is able to detect target parasite ova in samples with low EPG counts and that diagnostic accuracy is minimally affected by low EPG counts for the target parasites.

The favorable qualitative results, i.e., sensitivity and specificity, for VETSCAN IMAGYST were complemented by quantitative outcomes, namely Lin’s CCCs of 0.924–0.978 for strongyle EPG counts and coefficients of 0.944–0.955 for *Parascaris* spp. EPG counts, indicating moderate-to-strong agreement between the algorithm’s results and an expert parasitologist’s analyses. Our results have added credibility in that the manual FEC counts were performed by expert analysts working in a parasitology laboratory under optimal conditions that minimized variability of outcomes.

The expert parasitologists performing the study’s manual assays noted that when the NaNO_3_ solution was used, there were sometimes movement and color shifts in the sample filtrate under the coverslip during the first 2 min after slide preparation. They also noted that crystallization occurred after ~ 15 min on the edges of slides prepared with NaNO_3_, potentially affecting accuracy of the assay or its interpretation. Crystallization of saturated salt flotation solutions is a disadvantage that has been observed by other parasitologists [18, 25]. These characteristics did not occur with Sheather’s sugar solution, perhaps because of the differences in chemical bonds and solubility of salt vs. sugar compounds [18, 26]. While there is no universal or perfect flotation solution for recovery of all parasite ova [18], either the NaNO_3_ or Sheather’s sugar solutions can be effectively used to prepare samples for VETSCAN IMAGYST analysis. However, mean sensitivity and specificity percentages for both target parasites favored Group 3 sample preparations versus those for Group 2, indicating that Sheather’s sugar solution is a better option for a fecal flotation solution for diagnosing the target parasites. This recommendation is consistent with findings by other investigators [2, 26].

No sector of veterinary medicine is more dependent on intestinal parasite diagnosis and management than equine practice. Horses have a lifetime susceptibility to strongyles, the most prevalent and economically important equine nematode [5, 19]. As a result, periodic FEC tests are a cornerstone of evidence-based monitoring to assess individual and community parasite burdens within a given equine population. The fecal egg count reduction test (FERCT), where FECs are obtained before and after antiparasitic treatment, is a widely used determinant of treatment efficacy and the degree of parasite resistance to specific treatments. In herd surveillance programs, FEC testing is also used to identify and manage clinically normal but high-shedding horses that can increase infection pressure within a herd.

Specialized equipment required for FEC testing and the time required for the procedure discourages regular FEC testing. Rather than basing parasite control programs on FEC testing, many simply resort to indiscriminate treatment of horses with anthelmintics, an expedient that drives over-treatment and the dire problem of anthelmintic resistance. For example, recent large-scale survey data (*n* = 380 herds) indicate that only a minority of US horse owners regularly use FEC testing. While most respondents dewormed multiple times a year with ivermectin or other anthelmintics, fewer than a fourth used FECs on an occasional basis, < 10% using them on a regular basis, and < 5% used FECRTs [27]. To mitigate this discouraging trend, equine practitioners now have access to Vetscan Imagyst, an automated equine fecal testing system that can potentially minimize training requirements, offset operator variability, and provide an alternative, reliable egg count method.

## Data Availability

No datasets were generated or analysed during the current study.
